# Mozzarella Cheese Stretching: A Minireview

**DOI:** 10.17113/ftb.59.01.21.6707

**Published:** 2021-03

**Authors:** Mônica Correia Gonçalves, Haíssa Roberta Cardarelli

**Affiliations:** 1 Federal University of Paraíba, Department of Food Engineering, Center of Technology, Campus I, Cidade Universitária, Av. Castelo Branco s /n, João Pessoa, PB, 58051-900, Brazil; 2 Federal University of Paraíba, Center of Technology and Regional Development, Campus I, Rua dos Escoteiros, s/n Mangabeira, João Pessoa, PB, 58058-600, Brazil

**Keywords:** pasta filata cheese, functional properties, stretching temperature, calcium content

## Abstract

Mozzarella cheese stretching is a thermomechanical treatment influenced by factors such as pH, acidity, stretching time and temperature. The aim of this minireview is to provide information about the stretching step and the effect of the main factors on the functional properties of mozzarella. The presented studies show that stretching under higher temperatures promotes more interactions in the protein matrix, and changes occur in the calcium balance throughout the storage period that influence water mobility, proteolysis and lead to changes in mozzarella properties. Therefore, the information presented in this minireview may facilitate the production of mozzarella cheese with specific functional properties.

## INTRODUCTION

Mozzarella cheese belongs to the group of pasta filata or stretched-curd cheese types, whose stretching gives the cheese a unique fibrous texture (*1*). The curd is stretched with hot water and then cooled down. Some parameters should be controlled during the cheese stretching process, such as the insoluble calcium content (*2*), pH and thermomechanical treatment.

The functional properties of mozzarella, such as the viscoelasticity, fluidity, elasticity and oil release during heating, depend on the processing conditions due to the impact of these conditions on the cheese microstructure and composition (*3*). According to Lamichhane *et al.* (*4*), cheese functionality can be modified by optimizing or modulating the initial production conditions, which impact the process control parameters.

Therefore, mozzarella cheese manufacturers need to understand the process conditions to better control the stability of the cheese functional properties, increase its shelf life and improve its product consistency and performance, which are modified throughout the refrigerated storage. Therefore, the aim of this minireview is to present the main factors that influence the stretching step and lead to changes in the cheese properties to provide information to facilitate the production of high-quality mozzarella cheese.

## MANUFACTURING TECHNOLOGY

Mozzarella cheese can be produced from cow’s or buffalo’s milk, although the production of cow’s milk mozzarella is the highest, and it is sold worldwide (*5*). Mesophilic (*Lactococcus lactis* ssp. *lactis* and *Lactococcus lactis* ssp. *cremoris*) and thermophilic (*Streptococcus salivarius* ssp. *thermophilus*, *Lactobacillus delbrueckii* ssp. *bulgaricus* and/or *Lactobacillus helveticus*) cultures can be used in the production of this type of cheese (*2*, *6*). The main role of the starter (mesophilic or thermophilic) culture is the production of sufficient lactic acid to convert the curd into a mass that will stretch in warm water (*1*). This state is achieved by the right combination of pH and calcium content in the curd at the time of stretching. Thermophilic cultures are globally used on a larger scale in the production of mozzarella for pizza than mesophilic cultures since they are more suitable for obtaining the desired moisture content (between 48 and 52%) (*2*).

The addition of organic acid to milk can also be used to manufacture mozzarella cheese. In this case, acidification of the milk leads to higher solubilization of colloidal calcium and a lower level of protein-associated calcium in the mozzarella cheese (*7*), which enables stretching to occur at a higher pH=5.5-5.7 (*8*). Therefore, the lower fraction of insoluble calcium increases the hydration of casein, resulting in an increase in moisture and melting of mozzarella cheese obtained by direct acidification (*9*). Thus, the cheese becomes suitable for use in pizzas immediately after it is produced (*2*), which is not the case when mozzarella is produced using starter cultures. In addition, a lower degree of browning also occurs in cheese obtained by direct acidification than in cheese obtained with starter cultures (*10*, *11*). This is due to the starter cultures that produce small peptides and amino acids able to react with residual sugars in the system when subjected to heating (*11*).

Pizza cheese manufactured using citric acid as pH regulator had higher calcium concentration and pronounced meltability and stretchability (*12*). Table 1 (*1*, *8*-*11*, *13*) compares differences between direct acidification and starter culture used in the processing of mozzarella.

**Table 1:** Differences between the manufacturing parameters used in the processing of mozzarella cheese

Processing variable	Manufacturing parameter		Reference
Starter culture	Direct acidification
pH	5.0 to 5.2	5.5 to 5.7	(*8*)
Ca_insoluble_ fraction	High	Low	(*9*)
Ca_total_ content	High	Low	(*13*)
Moisture content	Low	High	(*13*)
Degree of browning	High	Low	(*10*, *11*)
Processing time	Long	Short	(*1*)

Mozzarella cheese production traditionally employs stretching in hot water, and an alternative method for its production is to stretch the curd after the addition of 1-1.5% melting or emulsifying salts consisting of citrates and phosphates before heating, and this process is followed by moulding. The cheese obtained with this method has improved melting, flavour and texture properties in addition to a greater recovery of milk constituents than the cheese produced with the addition of cultures and stretching in hot water (*14*).

### Mozzarella cheese stretching

The heat transfer during stretching must occur at a rate sufficient to transform the curd into a plastic and flowable consistency before it is kneaded and texturized. Plasticization and stretching are governed by the casein-associated calcium content at the time of stretching, which in turn is modulated by the total calcium content and the curd pH. Stretching is a thermomechanical treatment involving the application of mechanical energy (in the form of shear stress) and temperature. There are two essential parameters that need to be controlled for optimal curd stretching. The first is that the curd needs to be sufficiently acidified and demineralized, and the second parameter is the heat transfer between the curd and the water or the brine during stretching (*13*).

**Fig. 1:**
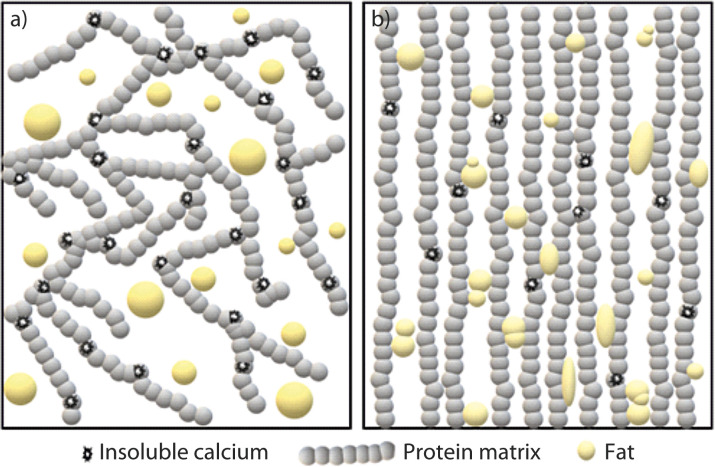
Schematic representation of changes in cheese structure: a) curd before thermomechanical treatment, and b) cheese after thermomechanical treatment

Traditionally, the curd is heated in hot water until it reaches the proper texture. This process imparts unique characteristics to the pasta filata cheese (*15*). When curd obtained by enzymatic coagulation and fermentation reaches a pH=5.4 to 5.2, dicalcium paracaseinate is converted to monocalcium paracaseinate, which favours fibre formation (*16*). The curd at the stretching pH has the ability to plasticize in hot water and reorganize into a unidirectional fibrous structure (*17*-*19*). In addition, increased protein matrix hydration occurs as the casein-associated calcium level decreases, which probably contributes strongly to curd plasticization (*8*). A schematic representation of the change in cheese structure due to the thermomechanical treatment can be seen in Fig. 1.

**Fig. 2:**
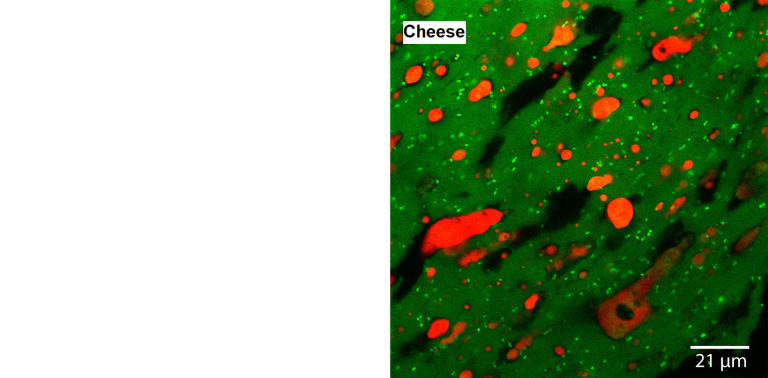
Microstructure of the curd before stretching at pH=5.2 and the cheese after the stretching. Reprinted with permission (*20*)

According to Gonçalves and Cardarelli (*20*), the protein matrix in curd in the stretching stage is more organised and porous, interwoven with fat globules of different sizes than the curd in previous manufacturing stages. At the end of the mozzarella cheese production, the protein matrix became more compact, with the agglomeration of more fat particles and with the incorporation of small individual fat globules. Fig. 2 (*20*) shows the microstructure of the curd at stretching stage (pH=5.2) and the cheese after the stretching stage.

As the curd temperature increases, there is a corresponding increase in the strength of the hydrophobic interactions within the protein matrix. Strengthening of the hydrophobic interactions results in the contraction of the protein matrix, forcing a small amount of water out and freeing the interstitial spaces around the fat globules. Therefore, the stretching is also responsible for the water distribution in the mozzarella cheese matrix, which differs from that of other cheese types (*11*).

### Curd structure reorganization

Several studies have indicated that the stretching step affects the mozzarella cheese microstructure (*3*, *21*, *22*). Therefore, the control of cheese properties requires an understanding of the position and interactions of various components, which is possible through studying the microstructure during processing and storage (*23*).

Cheese undergoes kneading and stretching during the thermomechanical treatment, which generates its typical fibrous appearance (*13*). Thermomechanical treatment forms fibrous anisotropic structures from the initial isotropic cheese curd (*24*). The stretching leads to the parallel alignment of the casein fibers, which are interspersed by water and fat channels (*13*), resulting in the formation of free serum (*25*) (Fig. 1). Microstructural images in the study by Sharma *et al.* (*22*) on mozzarella cheese showed that the fibrous appearance disappeared and a homogeneous structure formed with a fine dispersion of fat particles in a weak protein network. Immediately after manufacturing, mozzarella cheese undergoes significant structural rearrangement, and the free water that occupies the interstitial area around the fat channels is absorbed into the protein matrix (*11*).

Certain techniques can be used to study the changes that occur during the mozzarella manufacturing, including nuclear magnetic resonance (NMR), which have been shown to be powerful methods of evaluating changes in the structure and mobility of mozzarella cheese components (*11*, *26*, *27*). In addition to NMR, confocal microscopy is a tool with great potential to improve the understanding of milk and cheese microstructure, including the size and shape of fat globules (*28*), and it has been successfully used to study the three-dimensional network of fat and protein in both cheese and curd (*29*).

During storage, the composition of water and fat channels changes as the protein absorbs the free water formed during curd stretching within the channels (*30*). These changes have been observed in studies that have evaluated the microstructure of mozzarella cheese (*3*, *21*, *31*).

Microstructural images captured during cheese maturation show a continuum of proteins containing intact fat and coalesced fat droplets. These observations together with the decrease in serum extracted during ageing support the hypothesis that the increase in paracasein volume is due to an increase in hydration, probably as a result of proteolysis and solubilization of insoluble calcium (*21*). The increase in water-binding capacity in the matrix is an important phenomenon because it affects the water-holding capacity and consequently the ability of the cheese to flow and stretch during heating (*32*).

## FACTORS THAT INFLUENCE THE STRETCHING OF MOZZARELLA CHEESE

The milk composition, its treatment, pH, acidity, calcium content, stretching conditions (stretching water temperature, stirring speed, mechanical treatment) and the type of stretching (manual or mechanical) are some of the factors that influence the stretching of mozzarella cheese. Studies have shown that changes in processing conditions, such as the pH at whey drainage, interact with changes in composition (*e.g.* calcium content of milk), which together influence the final properties of cheese (*7*, *33*).

### Influence of milk composition

There are considerable individual changes in the concentrations of fat, protein, casein, lactose and minerals associated with seasonality. These changes, in turn, affect the gelation characteristics of the rennet and its cheese-making properties. In addition to composition, seasonality also affects the state (quality) of milk protein and fat and its standardization for cheese processing (*14*, *34*).

According to a study by Gonçalves and Cardarelli (*35*) with mozzarella cheese, the standardization of the milk casein/fat ratio used in cheese processing resulted in lower fat content, increased hardness and lower melting capacity because this relation is closely linked to the protein structure of the cheese. The casein/fat ratio is important because it influences the texture (*36*).

### Influence of raw or pasteurized milk

The chemical composition, and microbiological and enzymatic quality of milk have a major impact on the quality and consistency of the cheese. Mozzarella is traditionally manufactured using raw milk (*37*).

The heat treatment of milk to process mozzarella cheese is a relatively new phenomenon due to the opening of big manufacturing industries of mozzarella cheese. There are still several dairies that do not treat the milk with heat for mozzarella production. Pasteurization of milk has become a step in cheese making due to a number of countries and governmental agencies having restrictions on the use of raw milk (*11*). Moreover, for mozzarella cheese that is to consumed fresh, pasteurization of milk is recommended because the stretching process does not always destroy pathogens (*37*).

According to Patel *et al.* (*38*) the heat treatment of milk at 72 °C to process mozzarella cheese produces soft-bodied cheese with superior sensory flavour and keeps its quality comparable to the cheese made from raw milk. Increasing the intensity of milk pasteurization (from 72 to 82 °C) significantly reduced the flowability and stretchability of the mozzarella cheese submitted to heating (*39*).

### pH and acidity

According to Rowney *et al*. (*40*), the optimal condition for the stretching of mozzarella cheese occurs when the pH is in the range of 5.2 to 5.3, whereas Maldonado *et al*. (*41*) reported that the optimum pH for stretching ranged from 5.2 to 5.5. Lowering the pH favours a change in the distribution of casein-associated calcium to the soluble state (*2*). At higher pH values (~5.4), the proportion of casein-associated calcium (colloidal calcium) is relatively higher than that at low pH (*42*).

At pH=5.2, the curd is usually transferred to the stretching unit and a large proportion of casein is dissociated from the micelles, thus forming a longitudinal microstructure. When the curd is stretched at pH=5.3, it has a more structured texture, both when fresh and during maturation, than the mozzarella cheese obtained from the curd stretched at pH=5.0 (*43*). This may be due to a higher level of calcium in the curd that has been stretched at a higher pH value (*3*). The development of acidity during mozzarella cheese manufacturing should be controlled so that the correct combination of total calcium content, pH and moisture content in the curd are achieved during stretching (*7*).

### Calcium

Calcium plays an important role during mozzarella cheese processing because it helps with coagulation and promotes crosslinking between casein micelles (*44*). When the serum is removed, insoluble calcium is retained after cutting the curd, while most of the soluble calcium is lost in the serum (*45*). The lower solubilization of calcium in cheese than in milk occurs due to changes in calcium solubilization in the protein matrix, which has a higher solids concentration and higher ionic strength (*9*).

Studies have shown that there must be an optimal casein-associated calcium (insoluble or colloidal calcium) content, which is normally achieved through solubilization of this mineral by acidification, so that curd stretching occurs during mozzarella cheese production. Calcium solubilization during mozzarella processing occurs due to a reduction in pH (*8*, *46*).

Insoluble calcium is available to form crosslinks in the paracasein matrix when the curd is heated (*19*). Two parameters determine the amount of casein-associated calcium during the stretching: (*i*) the total calcium content in the curd, and (*ii*) the distribution of total calcium between the soluble and colloidal forms (*2*). As insoluble calcium content decreases, casein-casein interactions are fewer in number and weaker in strength, exhibiting a *para*-casein network with lower rigidity (*8*) and less thermal energy to obtain the gel-sol transition is needed (*47*).

### Type of stretching

Originally, mozzarella cheese was manually stretched (*48*), and this process is still performed in small industries. Hot water (70-80 °C) is added to the curd in pots, which is worked with the aid of large forks that move the curd along the wall of the pots, and the curd is kneaded, melted and stretched (*49*).

Mozzarella manufacturers use a wide range of industrial equipment for processing pasta filata cheese, including equipment with batch or continuous processes and single or double screw systems designed with different materials, geometries and heating systems, in which the process control parameters, such as the temperature, curd feed rate and screw speed, can be adjusted. Consequently, thermomechanical treatments may differ considerably depending on the used equipment (*21*). Mozzarella cheese can also be stretched using extruders, a method that is gaining significant commercial acceptance (*13*). In this system, the plasticization (gel-sol transition) is influenced by the stretching temperature, residence time and curd composition, especially by the calcium content (*50*).

Mechanical mixers with one or several screws are used to stretch the curd in hot water at temperatures controlled by steam injection (*2*, *22*), and the hot water usually ranges from 82 to 85 °C (*51*). An advantage of the mechanical treatment is that individual curd particles are rapidly transformed into a heterogeneous but continuous flowable mass that can be easily moulded (*15*). However, this treatment also causes a heterogeneous distribution of moisture in the mozzarella cheese (*30*), and increased stirring speeds result in higher fat loss and lower moisture content (*52*).

With the objective of minimizing such problems, the development of systems for stretching mozzarella cheese without using water has been studied. In this system, heating can be provided by direct steam injection, electromagnetic energy, heated auger bodies or a combination of these (*53*). However, there is still a lack of studies dealing with equipment sizing or setting parameters based on the physical properties of cheese curd during the main stages of plasticization, conveying and texturization (*48*).

## EFFECTS OF STRETCHING CONDITIONS ON THE QUALITY AND FUNCTIONAL PROPERTIES OF MOZZARELLA CHEESE

The temperature of the stretching water varies from 60 to 85 °C, and the temperature of the cheese as it leaves the mixers ranges from 50 to 65 °C (*54*). According to Fox *et al*. (*55*), the curd is heated in hot water (70-75 °C), kneaded and stretched when the pH reaches approx. 5.4. If the stretching temperature is below the sol-gel transition temperature, which is also called the plasticizing temperature, the fibrous structure typical of the curd will not be obtained (*56*). Regardless of the adopted type of stretching (*i.e.* manual or mechanical), the use of curd milling and cutting machines prior to stretching is recommended (*57*).

Table 2 (*49*, *51*, *54*, *55*, *58*-*60*) shows different stretching conditions employed in mozzarella cheese processing. The literature reports stretching water temperatures of 50 to 85 °C and pH ranging from 5.0 to 5.5. The variations in temperature and pH depend on the conditions used in the mozzarella stretching and influence the cheese composition and thermophysical properties.

**Table 2:** Stretching conditions used in mozzarella cheese processing

*t*_stretching_/°C	pH	Type of stretching	Reference
70-80	4.8-5.3	Manual	(*49*)
82 70-80	5.0-5.3	Mechanical Manual	(*51*)
65-85	5.3	Mechanical	(*54*)
55-65	5.3	Mechanical	(*58*)
70-75	5.4	-	(*55*)
50-80	5.0-5.5	-	(*59*)
55-70	5.2	-	(*60*)

- not cited in the reference

The curd stretching temperature influences the viability and activity of the starter bacteria in the final cheese. For this reason, a higher stretching temperature is sometimes desirable to increase the shelf life as it promotes greater thermal inactivation of microorganisms (*61*). Yun *et al*. (*62*) reported that both *Streptococcus thermophilus* and *Lactobacillus delbrueckii* ssp. *bulgaricus* survived and remained metabolically active when stretching was performed with water at low temperature (55 °C in the centre of the curd). Depending on the extent of heat inactivation during stretching, the activity of the residual coagulant in low-moisture mozzarella cheese may also vary (*63*). According to Yun *et al*. (*64*), primary and secondary proteolysis of mozzarella decreased when the stretching water temperature was increased from 62 to 66 °C.

The stretching temperature to which cheese is subjected influences the structure of cheese as it affects its components and their interactions, including changes in the physical state of fat and the molecular interactions between casein and fat (*4*). A difference in the stretching temperature of only a few degrees within the critical range of 60 to 65 °C drastically affects the cheese properties (*32*). According to a study by Renda *et al.* (*52*) increasing the stretching temperature from 55 to 75 °C reduced the elasticity of the mozzarella cheese and resulted in an increase in fat globule size and free oil content. According to Rowney *et al*. (*31*), these changes indicated that the aggregation and coalescence of fat globules during the cooking and stretching process determine the amount of free oil in mozzarella.

The curd temperature during the mozzarella cheese stretching is generally between 55 and 65 °C (*57*). At these temperatures, a corresponding increase in the strength of the hydrophobic interactions occurs within the protein matrix as the curd temperature increases, and this strengthening of the hydrophobic interactions results in the contraction of the protein matrix as the hydrophobic regions inside the protein get closer, which forces some of the water to change into a free state in the interstitial spaces around the fat globules (*65*).

A study of mechanical stretching demonstrated that increasing the stretching water temperature from 57 to 74 °C at a constant stirring speed of 12 rpm resulted in a shorter residence time in the machine, a higher cheese temperature when leaving the equipment, less mechanical work during stretching, lower fat loss in the stretching water and cheese with a higher fat content on a dry basis immediately after manufacturing (*64*).

The effect of the kneading time (180, 420 and 600 s) and the temperature of the stretched curd (55, 60 and 70 °C) on the chemical composition and yield of the cheese was investigated with a mozzarella stretching machine model system. An increase in temperature from 55 to 70 °C reduced the cheese yield from 0.88 to 0.59 g/g compared to the initial mass of the curd at constant temperature, and the yield also decreased with increased stretching time (*55*).

According to Banville *et al*. (*21*), the size and distribution of the fat globules are influenced by the stretching conditions. Smaller fat globules were observed in the cheese subjected to lower temperatures and lower stretching time, whereas larger fat globules and fat globule aggregation in the protein cavities in addition to the presence of free fat were observed in the cheese subjected to more severe stretching conditions. These results showed that the amount of mechanical energy supplied was directly proportional to the fat loss in the cheese and the amount of free whey was related to the thermal treatment intensity. The authors concluded that thermomechanical systems impacted the cheese composition, loss of solids and microstructure.

The stretching process is a heat treatment that profoundly affects the cheese composition and proteolysis during mozzarella storage. According to a study by Mulvaney *et al*. (*48*), increasing the stretching temperature from 55 to 75 °C reduced the mozzarella cheese elasticity, thereby resulting in increased fat globule size and free oil content. Rowney *et al*. (*31*) predicted that the aggregation and coalescence of fat globules during cooking and stretching determines the amount of free oil in mozzarella. Excessive and limited oil release are both considered defects (*16*) because they influence the appearance, taste and texture of mozzarella cheese when subjected to heating.

Traditionally mozzarella requires a maturation period between 7 and 21 days to develop optimal organoleptic and baking qualities (*53*). During this period the distribution between casein-associated calcium (insoluble calcium) and soluble calcium is crucial for the functionality of mozzarella cheese (*66*, *67*). A study by Joshi *et al*. (*46*) revealed that insoluble calcium plays an important role in improving the melting and other functional properties of mozzarella and the proportion of insoluble calcium and soluble calcium continues to change slowly. A partial solubilization of insoluble calcium during the first stage of maturation likely occurs due to a pseudobalance between the soluble and insoluble forms of calcium in the cheese (*47*). The solubilization of insoluble calcium during mozzarella cheese storage has been previously reported (*66*-*68*), and these changes are known to be of paramount importance for the hydration of the paracasein matrix and lead to changes in cheese texture during the first 10 days of storage (*66*).

Therefore, several strategies have been adopted for altering the calcium balance during cheese production. In milk, changes in the fraction of insoluble calcium in total calcium have been made through preacidification, the addition of calcium chelating agents and acidification control. In cheese, the calcium content can be altered by modifying the process conditions (*45*).

Cheese with high total calcium content has a lower melting capacity than the cheese with a low total calcium content according to Lucey and Fox (*18*). The concentration of total calcium in the cheese is one of the main factors that contribute to its melting because most of the calcium in the cheese is in an insoluble form that is capable of crosslinking with casein, involving phosphoserine groups (*46*). These crosslinks help reinforce the entire protein network, thereby promoting greater stiffness and when heated, the melting of the cheese decreased (*46*, *69*). A decrease in calcium mass fraction from 0.65 to 0.48, 0.42 and 0.35% increases the cheese melting capacity by 1.4-, 2.1- and 2.6-fold, respectively, because of the reduction in the crosslinks between the casein micelles of the cheese matrix, which make the cheese softer and easier to melt (*44*).

Lucey and Fox (*18*) have suggested that insoluble calcium plays a much more significant role in determining the cheese textural properties than the total calcium content. According to Fathollahi *et al*. (*70*), the decrease in insoluble calcium reduces the electrostatic interactions between caseins and leads to a more open protein matrix, thus making the casein molecules more susceptible to proteolysis.

The insoluble calcium content plays an important role in the melting of skim mozzarella cheese. Joshi *et al*. (*46*) found that the decrease in insoluble calcium promoted an increase in melting. In another study, part-skim mozzarella cheese was obtained from the preacidification of milk, and cheese with a lower calcium content had a more homogeneous structure, a more hydrated protein matrix, a higher number of fat particles and higher emulsification, which resulted in increased melting of this cheese (*44*). According to McMahon *et al*. (*71*), the low calcium content in fat-free mozzarella cheese results in a homogeneous and less dense protein structure than the cheese with the normal calcium content, thus producing a softer cheese with an increase in melting capacity independent of the pH and moisture content. Table 3 (*18*, *21*, *31*, *44*, *46*, *52*, *55*, *69*, *72*, *73*) summarises the processing variables and their impact on cheese functional properties.

**Table 3:** Processing variables and their impact on mozzarella cheese functional properties

Processing variable	Impact on functional properties	Reference
Stretching temperature*	Elasticity** Fat globule size* Free oil content* Fat coalescence* Cheese yield**	(*52*) (*52*) (*52*) (*31*) (*55*)
Stretching temperature and time*	**Fat globule size	(*21*)
Total calcium*	Melting capacity**	(*18*, *44*, *46*, *69*, *73*)
Insoluble calcium*	Melting capacity**	(*46*, *72*)

*increase, **decrease

According to the discussed studies, the processing variables are not independent and have complex interactions. If some aspect of the composition is changed, *e.g.* calcium content, inevitably others will be changed, *e.g.* meltability. In addition, changes in processing conditions, for example lowering the pH, interact with changes in composition, for example calcium content, having a combined and complex effect on the functional properties of mozzarella cheese (*7*, *33*).

## INFLUENCE OF COOLING OF STRETCHED MOZZARELLA ON ITS FUNCIONALITY

After the stretching stage, the hot curd is subjected to sufficient pre-cooling to maintain its shape when removed from the mould and immersed in cold brine. When cooled in brine, low moisture mozzarella blocks should be kept in brine long enough to cool to an internal temperature of 6 °C or less. Insufficient cooling of the blocks before being vacuum-packed or stacked without packaging means that the cheese will be internally hot, with excessive protein breakdown and continuous acid development occurring if the salt content is also low, 1% or less (*1*).

Physicochemical changes of high-moisture mozzarella cheese during frozen storage and subsequent refrigerated storage (after thawing) were evaluated with NMR relaxometry and casein dehydration related to freezing was observable through changes in the water relaxation times within the matrix, which were confirmed by microstructural observations that showed the formation of larger serum channels surrounded by the protein matrix and formation of relatively bigger fat globule clusters in the samples submitted to freezing than in fresh cheese (*73*). The formation of larger ice crystals during storage because of crystal growth and recrystallization can promote microstructural changes and the disruption of the casein matrix (*74*).

Alinovi and Mucchetti (*75*) studied the eﬀects of two freezing/thawing methods (the presence or absence of a covering liquid during the process) with high-moisture mozzarella cheese. The cheese processed with a covering liquid had longer freezing times and showed water absorption phenomena during thawing. Freezing may also be effectively applied to control or extend the functional shelf-life of low-moisture part-skim mozzarella cheese shipped to long-distance markets (*76*).

## INFLUENCE OF PROTEOLYSIS DURING STORAGE ON THE FUNCIONALITY OF MOZZARELLA CHEESE

According to a study by Gonçalves and Cardarelli (*77*), the change in the stretching temperature (from 75 to 85 °C) did not aﬀect the cheese composition, but mainly promoted changes during the refrigerated storage time.

Proteolysis occurs through the action of residual enzymes present in cheese that hydrolyze casein, causing a breakdown in the protein matrix. However, the heating that occurs during stretching reduces the activity of the residual rennet in the curd, which reduces the extent of primary proteolysis during the storage of the mozzarella cheese (*78*).

Proteolytic changes in mozzarella curd influence melting, stretching and fat leakage characteristics (*79*). Gonçalves and Cardarelli (*77*) reported that there was a decrease in water mobility and an increase in the electrostatic interactions and hydrogen bonds with higher intensity in the mozzarella cheese subjected to water stretching at 85 °C, which was reflected in lower casein degradation peaks observed in capillary electrophoresis than in the cheese stretched with water at 75 °C.

## CONCLUSIONS

Stretching is a key step for determining the cheese characteristics after manufacturing and during storage. However, a few studies have separately evaluated the factors influencing the stretching process or stretching temperature by focusing on the reactions involved in the stretching step as well as the impact of these reactions on the functional properties of mozzarella throughout the refrigerated storage. A wide range of stretching water pH (4.8 to 5.5) and temperature (50-85 °C) values is used in mozzarella manufacturing. Stretching under higher temperatures promotes more protein interactions, influences the viability and activity of the starter bacteria in the cheese, composition, yield, melting, water mobility, proteolysis and globule size, which may impact the release of oil in the cheese. Throughout the storage period, the fraction of calcium is changed, and these changes influence the water mobility in the protein matrix. This review provides information that may assist manufacturers in controlling the proteolysis, texture and functional properties, such as melting and oil release, of mozzarella cheese.
